# Transcription, Alternative Splicing, and Post-Translational Regulation of CaLOXs in the Dynamic Regulation of Jasmonate Levels in Wounded Pepper Leaves

**DOI:** 10.3390/plants15010045

**Published:** 2025-12-23

**Authors:** Juliette T. Keith, Yinting Chen, Jennifer Gabriel, Nicole M. van Dam, Jacqueline C. Bede

**Affiliations:** 1 Department of Plant Science, McGill University, 21,111 rue Lakeshore, Ste-Anne-de-Bellevue, QC H9X 3V9, Canada; juliette.keith@mail.mcgill.ca (J.T.K.); yinting.chen@mail.mcgill.ca (Y.C.); 2 German Centre for Integrative Biodiversity Research (iDiv), Halle-Jena-Leipzig, Deutscher Platz 52, D-04103 Leipzig, Germany; jennifer.gabriel@idiv.de (J.G.); vandam@igzev.de (N.M.v.D.); 3 Institute of Biodiversity, Ecology and Evolution (IBEE), Friedrich Schiller University Jena, Dornburger Straße 159, D-07743 Jena, Germany; 4 Leibniz Institute of Vegetable and Ornamental Crops (IGZ), Theodor-Echtermeyer Weg 1, D-14979 Großbeeren, Germany

**Keywords:** alternative splicing, jasmonate, post-translational modification, transcription, wounding

## Abstract

In response to stresses, jasmonates increase rapidly, leading to plant resistance against necrotrophic pathogens and chewing insect herbivores. Jasmonate biosynthesis is regulated at many levels, including transcriptionally, through alternative splicing, and the phosphorylation of the 13*S*-lipoxygenase (LOX) that catalyzes an early step in jasmonate biosynthesis. In pepper, transcriptomic analysis of a foliar wounding time course was conducted to deepen our understanding of these regulatory mechanisms. All four *CaLOX*s are constitutively expressed. ***CaLOX2***, which encodes an enzyme with a Ser in a predicted regulatory phosphosite, shows a rapid but short-lived increase in wound-induced expression. In contrast, ***CaLOX7***, which encodes a protein with a non-phosphorylatable Ala at the phosphosite, shows higher wound-induced expression at 6 h. As well, at this timepoint, there is a predicted increase in exon 4 retention in ***CaLOX8*** transcripts in wounded plants. ChimeraX protein modeling predicts that the retention of exon 4 may negatively affect enzyme activity, possibly by blocking access to the enzyme’s active site. The transcription, alternative splicing, and post-translational regulation of CaLOX enzymes support the dynamic fluctuations observed in the jasmonates, which increase rapidly upon wounding and return to basal levels at 6 h post-stress.

## 1. Introduction

Jasmonates are a family of oxylipin signaling molecules that regulate plant development processes, including flowering time and senescence [1,2,3], and stress responses [4,5,6]. In response to mechanical damage, such as that incurred by chewing insect herbivory, jasmonate signaling gives rise to approximately 95% of the wound-induced protein synthesis that leads to the production of protective plant specialized metabolites and morphological barriers [7,8]. Given the importance of this pathway in plant resistance to pathogens and insect herbivores, understanding its regulation is key and may lead to novel strategies to enhance crop productivity [9].

After wounding or recognition of a necrotrophic pathogen, jasmonate biosynthesis starts in the chloroplast, where *α*-linolenic acid, a substrate of lipoxygenases (LOXs), is released from the galactolipids of the chloroplast membrane by a galactolipase [10]. The fatty acid undergoes oxidation, catalyzed by a member of the 13*S*-LOX class of lipoxygenases, producing (13*S*)-hydroperoxy-octadecatrienoic acid (13-HPOT) [11]. At this point in the pathway, different oxylipins may be produced. The reaction that converts 13-HPOT to (12*S*-13*S*)-epoxy-hydroperoxy-octadecatrienoic acid ((12*S*-13*S*)-EOT) is catalyzed by allene oxide synthase (AOS) and commits the pathway towards jasmonate biosynthesis [12]. (12*S*-13*S*)-EOT is reconfigured by allene oxide cyclase (AOC) into the more stable (9*S*,13*S*)-12-*oxo*-phytodienoic acid (OPDA), a bioactive jasmonate [13,14,15]. OPDA is transported from the chloroplast into the cytosol and then into the peroxisome [16,17]. In this organelle, jasmonate biosynthesis continues with the reduction of OPDA by OPDA reductase 3 (OPR3) [18,19,20]. After esterification to CoA [21], OPC-8 undergoes *β*-oxidation by the fatty acid *β*-oxidation machinery composed of acyl-CoA oxidase (ACX), multifunctional protein (MFP), and 3-ketoacyl CoA thiolase (KAT) [22,23,24,25,26,27]. The product, jasmonoyl-CoA, is transformed into jasmonic acid (JA), which moves into the cytosol [28,29,30]. There, it may be converted into its biologically active form, jasmonoyl-isoleucine (JA-Ile), by jasmonate resistant 1 (JAR1) [31].

After entering the nucleus [32], JA-Ile binds to the Skp1/Cullin1/F-box protein coronatine-insensitive 1 (SCF^CO1^) complex, bridging the ubiquitin E3 ligase with its target repressor jasmonate ZIM-domain (JAZ) proteins, leading to its degradation through the 26*S*-proteasome, thus freeing MYC2, MYC3, or MYC4 transcription factors from repression and allowing JA-Ile-responsive gene expression to proceed [32,33,34,35,36]. Targets of these transcription factors include genes that encode enzymes in the jasmonate biosynthetic pathway, leading to feedforward regulation [37].

In unwounded leaves, the basal JA level in *Arabidopsis thaliana* (arabidopsis) is less than 30 pmol/gm fresh mass [38]. After wounding, JA levels increase within seconds after mechanical damage, reaching levels that are over 10-fold higher than those of unwounded leaves within 2 min. This response is too rapid to be attributed to the increase in the expression of genes encoding enzymes in the jasmonate biosynthesis pathway. This indicates that the pathway must be constitutively present and activated upon damage [10,39,40]. Thivierge et al. [41] found that AtLOX2, which catalyzes an early step in jasmonate biosynthesis, is constitutively phosphorylated on Serine^600^ (Ser^600^) and dephosphorylated in response to wounding. Using phosphovariants, in vitro studies confirmed that the unphosphorylatable AtLOX2 had over a 100 times increase in V_max_ compared to the phosphomimic, which likely reflects blocking of the substrate from the catalytic site [42]. Therefore, wounding stress is proposed to lead to the dephosphorylation of AtLOX2, contributing to the rapid increase in JA-Ile biosynthesis.

In arabidopsis AtLOX2, the phosphosite, where the Ser^600^ is shown in red, is flanked by the amino acid sequence ARQ**S**LVNG [42]. The sequence ARQ**S**LVNG is relatively conserved among plant 13*S*-LOXs; however, there are variations at the phosphosite Ser, where Ala or Ile occupy this position, generating non-phosphorylatable variants. We chose pepper (*Capsicum annuum*) as our study organism since pepper has four 13*S*-LOXs [43]; **CaLOX2** and **CaLOX8** have a Ser at the phosphosite, whereas **CaLOX6** and **CaLOX7** have an unphosphorylatable Ala at this site, suggesting that these proteins may be regulated at the post-translational or transcriptional levels, respectively (Note: Pepper LOXs have been color-coded; those with an Ala at the phosphosite (**CaLOX6** and **CaLOX7**) in orange and those with a Ser at this site (**CaLOX2** and **CaLOX8**) in blue).

Alternative splicing (AS) also plays an important role in post-transcriptional regulation of stress-related gene expression in plants [44]. In tea, *Camellia sinensis*, six out of eleven LOXs have splice variants [45]. The splice variants of CsLOX3 and CsLOX1 contained premature stop codons, suggesting they are targeted for nonsense-mediated decay, while the splice variants of CsLOX2, CsLOX5, CsLOX9, and CsLOX10 are proposed to encode truncated proteins without specific functional domains. For example, AS of CsLOX5 and CsLOX10 removes the oxygen-binding domain of these enzymes, producing truncated proteins without an oxygenase function. These nonactive splice variants are proposed to interfere with functional LOXs by competing for transcriptional machinery or for substrates at the protein level. The splice variants in tea do not appear to affect the predicted phosphosite based on the AtLOX2 amino acid sequence.

As previously mentioned, JAZ proteins are negative regulators of jasmonoyl-isoleucine-responsive gene expression [33,34,35]. As a means of negative feedback regulation, genes encoding these proteins are induced in response to wounding [8]. Splice variants of JAZ proteins desensitize the plant to jasmonate signaling [46,47]. These splice isoforms are able to bind to the MYC transcription factors; however, they lack the C-terminal jas motif and, thus, cannot be targeted for degradation, even in the presence of high JA-Ile levels.

The above highlights that there are possible multiple levels of regulation that shape jasmonate dynamics upon wounding; however, the underlying mechanisms are not fully understood. In this study, we are interested in the transcriptional expression of genes encoding jasmonate biosynthetic enzymes, the expression pattern of *LOX* genes that encode either a Ser or Ala at the regulatory phosphosite in the mature protein, and alternative splicing events of *LOX* and *JAZ* genes. Thus, we conducted a time course of wound responses in pepper leaves to deepen our understanding of the regulation of the jasmonate pathway.

## 2. Results

### 2.1. Phytohormone Levels in Wounded Pepper Leaves

**Figure 1:**
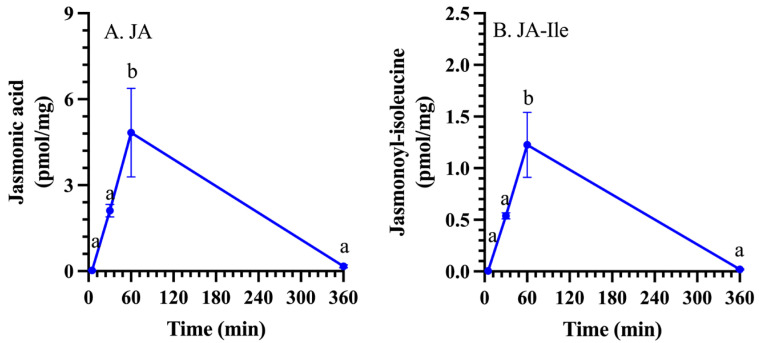
Foliar jasmonate levels increase rapidly and dynamically after wounding. At the four true leaf stage, individual pepper (*Capsicum annuum*) plants were wounded (*n* = 4) on the second set of fully unfurled leaves using a hole-punch, removing approximately 12% of the leaf, avoiding the midvein. Foliar jasmonate levels over a 6 h time course were measured by ultrahigh-performance liquid chromatography–mass spectrometry (UPLC-MS). (**A**) Jasmonic acid (JA), (**B**) Jasmonoyl-isoleucine (JA-Ile). Alphabetical letters indicate significant differences in wounded plants determined by univariate analysis of variance (one-way ANOVA) followed by Tukey honestly significant difference (HSD) *post hoc* test (*p* ≤ 0.05) (Supplemental Table S1).

Foliar JA and JA-Ile levels rose sharply in the first hour after wounding and then returned to basal levels at 6 h post-damage (Figure 1, Supplemental Table S1).

### 2.2. Wound-Induced Gene Expression in Pepper Leaves

After processing and removal of poor-quality reads, an average of 103.97 million 100 bp reads per sample was obtained with a Pfred score of 39 or above (Supplemental Table S2). The high-quality reads aligned with the NCBI *C. annuum* genome assembly UCD10Xv1.1 (48,341 annotated genes) with an average mapping efficiency of 91.6% uniquely mapped reads. One thousand three hundred and forty-five (1345) genes, representing 5.3% of the total expressed genes, were wound-responsive at one or more timepoints over the experimental time course; of these, 1141 genes (84.8%) and 205 genes (15.2%) were upregulated or downregulated, respectively, in wounded leaves (Supplemental Figure S1).

**Figure 2:**
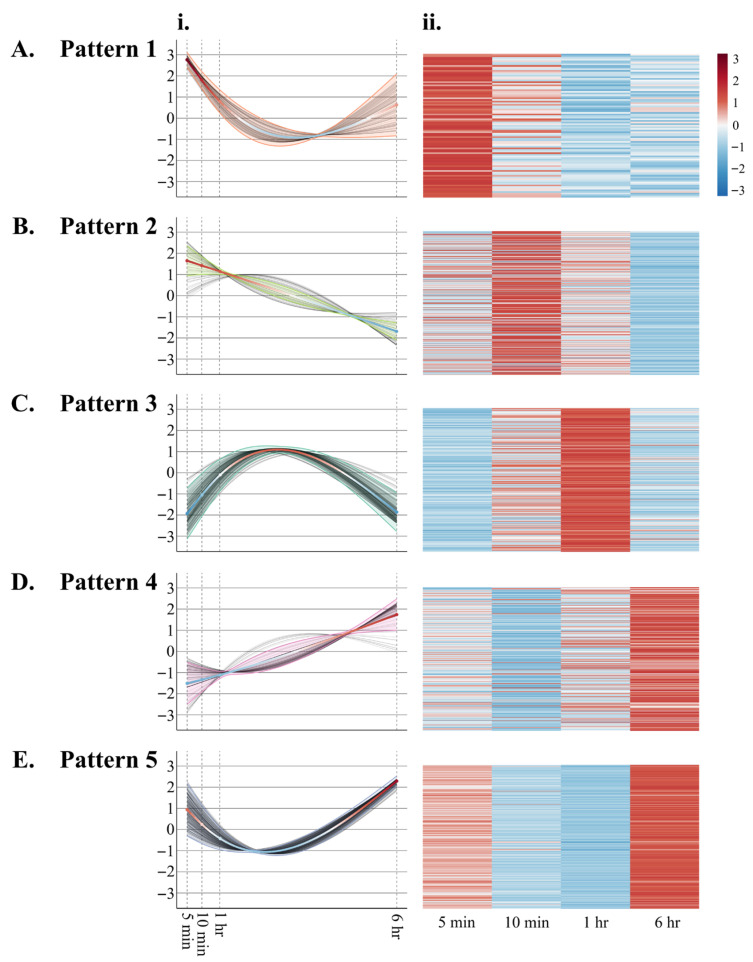
Foliar wound-induced gene expression clusters into five patterns. At the four true leaf stage, individual pepper (*Capsicum annuum*) plants were either left undamaged (*n* = 4) or wounded (*n* = 4) on the second set of fully unfurled leaves using a hole-punch, removing approximately 12% of the leaf, avoiding the midvein. Foliar gene expression over a 6 h time course was measured by RNA-Seq (Supplemental Table S3A,B). Wound-induced genes identified by DESeq2 (Padj ≤ 0.05) clustered into 5 patterns: (**A**) Pattern 1: Repressed gene expression after wounding, (**B**) Pattern 2: Highest expression at 30 min post-wounding, (**C**) Pattern 3: Highest expression at 1 h post-wounding, (**D**) Pattern 4: Highest expression at 6 h post-wounding, and (**E**) Pattern 5: Dynamic expression initially decreasing and then increasing. Data are represented by (i) smoothing spline plots and (ii) heatmaps. Red in heatmaps indicates highly expressed genes.

Wound-responsive genes clustered into 5 main patterns (Figure 2, Supplemental Table S3): Pattern 1: Genes that show a repressed gene expression after wounding; Pattern 2: Genes that show highest expression at 30 min post-wounding; Pattern 3: Genes that show highest expression 1 h post-wounding; Pattern 4: Genes that show highest expression 6 h post-wounding; and Pattern 5: Genes that show dynamic expression initially decreasing but then showing the highest expression at 6 h post-wounding.

### 2.3. Wound-Induced Expression of Genes Encoding Enzymes in Jasmonate Biosynthesis in Pepper Leaves

**Figure 3:**
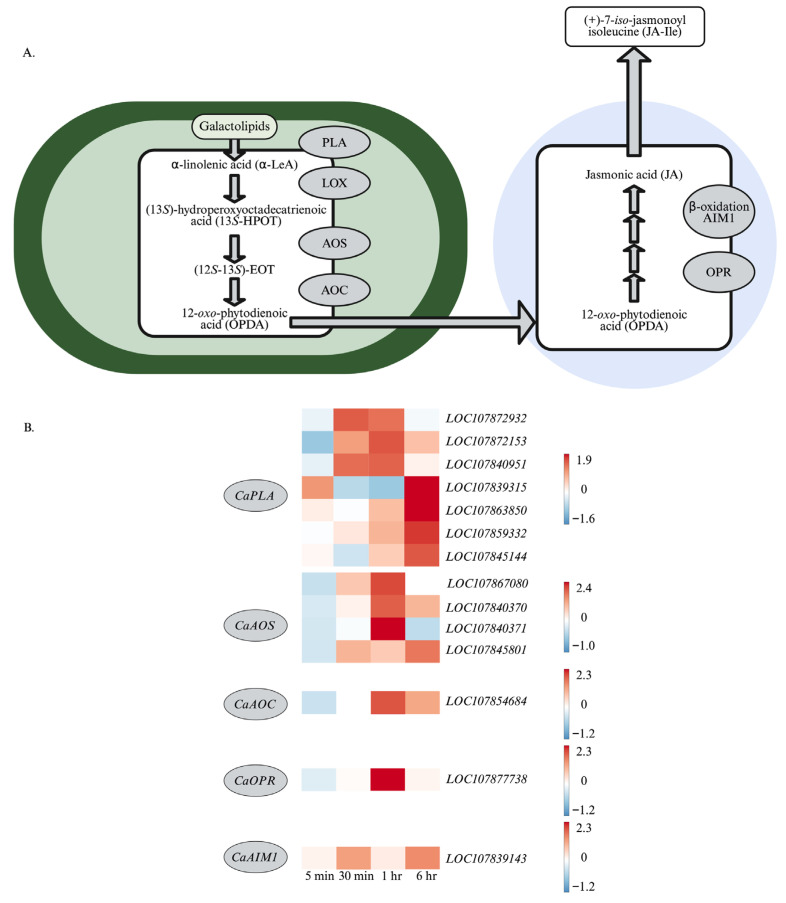
Genes encoding enzymes in the jasmonate pathway are wound-induced. At the four true leaf stage, individual pepper (*Capsicum annuum*) plants were either left undamaged (*n* = 4) or wounded (*n* = 4) on the second set of fully unfurled leaves using a hole-punch, removing approximately 12% of the leaf, avoiding the midvein. Foliar gene expression over a 6 h time course was measured by RNA-Seq, and wound-induced genes were identified by DESeq2 (Padj ≤ 0.05). (Supplemental Table S3A,B). (**A**) General jasmonate biosynthetic pathway; (**B**) Heatmaps visualizing wound-induced expression of genes encoding jasmonate biosynthetic enzymes. Heatmaps represent the Z-score, with red indicating highly expressed genes. Abbreviations: phospholipases (PLA), lipoxygenase (LOX), allene oxide synthase (AOS), allene oxide cyclase (AOC), (12*S*-13*S*)-epoxy-octadecatrienoic acid ((12*S*-13*S*)-EOT), 12-*oxo*-phytodienoic acid reductase (OPR). Figure created with BioRender (https://www.biorender.com/).

Within 30 min after wounding, early and sustained expression of genes encoding early enzymes in the jasmonate biosynthetic enzymes, including chloroplastic phospholipases (phospholipase A1 (LOC107872932), CaDAD1 (LOC107872153), CaPLIP2 (LOC107840951)) and allene oxide synthase1 (CaAOS1 (LOC107867080)) (Figure 3). At one hour post-wounding, genes encoding CaAOS3 (LOC107840370, LOC107840371), allene oxide cyclase (CaAOC (LOC107854684)), and 12-*oxo*-phytodienoic acid reductase3 (CaOPR3 (LOC107877738)) are strongly expressed. Later wound-induced jasmonate biosynthetic genes include *CaAOS3* (*LOC107845801*), peroxisomal *CaAIM1* (*LOC107839143*), and chloroplastic *phospholipases* (*LOC107839315*, *LOC107863850*, *LOC107859332*), including *CaFAR2* (*LOC107845144*).

**Figure 4:**
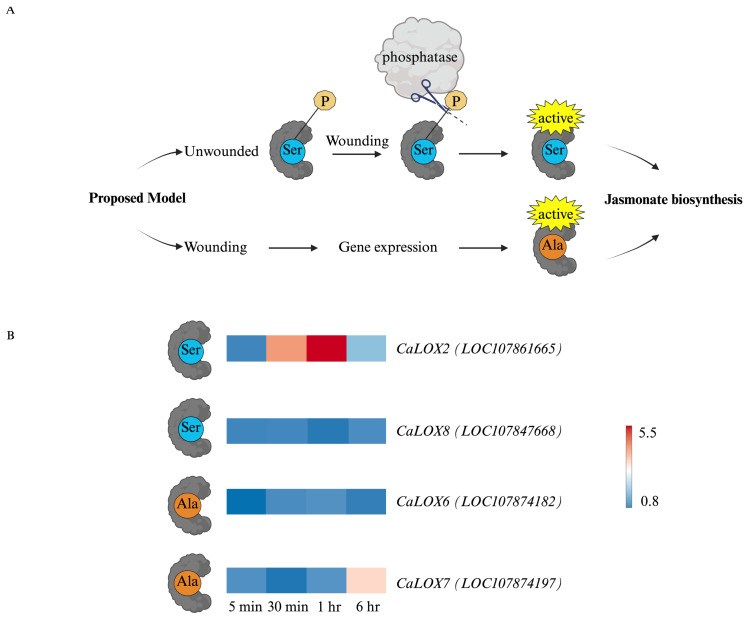
Genes encoding pepper 13*S*-lipoxygenases are differentially expressed temporally after wounding. At the four true leaf stage, individual pepper (*Capsicum annuum*) plants were either left undamaged (*n* = 4) or wounded (*n* = 4) on the second set of fully unfurled leaves using a hole-punch, removing approximately 12% of the leaf, avoiding the midvein. Foliar gene expression over a 6 h time course was measured by RNA-Seq, and wound-induced genes were identified by DESeq2 (Padj ≤ 0.05) (Supplemental Table S3A,B). (**A**) Proposed model for the regulation of jasmonate biosynthesis by *CaLOX* gene expression; genes encoding CaLOX with a **Ser** at the proposed phosphosite (color-coded in blue) are expressed early after wounding and are likely regulated through post-translational phosphorylation, whereas genes encoding a CaLOX with an **Ala** at the phosphosite (color-coded in orange) are expressed later after wounding. Pepper **CaLOX6** and **CaLOX7** have an **Ala** at the predicted phosphosite, whereas **CaLOX2** and **CaLOX8** have a **Ser**. (**B**) Heatmaps visualizing expression of genes encoding 13*S*-LOXs: ***CaLOX2*** (*LOC107861665*), ***CaLOX8*** (*LOC107847668*), ***CaLOX6*** (*LOC107874182*), and ***CaLOX7*** (*LOC107874197*). As LOXs have circadian rhythms [48], the ratio of gene expression in wounded over undamaged plants is shown. Red in heatmaps indicates highly expressed genes. Abbreviations: Alanine (Ala), lipoxygenase (LOX), serine (Ser). Figure created with BioRender (https://www.biorender.com/).

Focusing on pepper LOXs, we were interested in the expression of genes that encoded proteins with a Ser or an Ala at the phosphosite, predicting that those with a Ser would be constitutively expressed or immediately after wounding, whereas those with an Ala would be expressed later [41,42]. All CaLOXs were constitutively expressed. ***CaLOX2***, which encodes an enzyme with a Ser at the phosphosite, shows rapid but short-lived expression (Figure 4). In contrast, ***CaLOX7***, which encodes an enzyme with Ala at the phosphosite, is highly expressed 6 h after wounding.

### 2.4. Alternative Splicing of 13S-CaLOXs in Wounded Pepper Leaves

**Figure 5:**
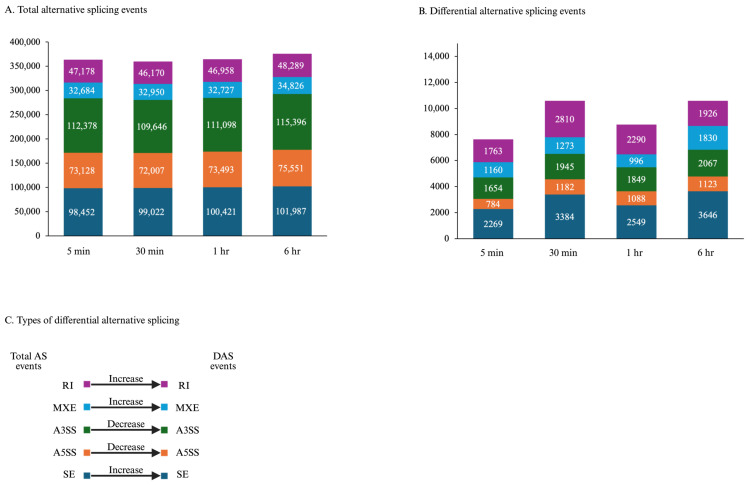
Alternative splicing events change by 2–3% in wounded pepper plants. At the four true leaf stage, individual pepper (*Capsicum annuum*) plants were either left undamaged (*n* = 4) or wounded (*n* = 4) on the second set of fully unfurled leaves using a hole-punch, removing approximately 12% of the leaf, avoiding the midvein. Foliar gene expression over a 6 h time course was measured by RNA-Seq (Supplemental Table S3A). Alternative splicing (AS) events were predicted by rMATs. (**A**) Total AS events at each timepoint in undamaged and wounded plants. (**B**) Differential AS (DAS) events between undamaged and wounded leaves. (**C**) Type of changes in AS between undamaged and wounded leaves. Abbreviations: Skipped exon (SE), alternative 5′ and 3′ splice site (A5SS, A3SS, respectively), mutually exclusive exons (MXE), and intron retention (RI). Figure created with BioRender (https://www.biorender.com/).

In addition to transcriptional regulation, genes may undergo alternative splicing, potentially resulting in novel proteins or nonfunctional proteins. In undamaged leaves, about 350,000 splicing events are predicted, with the majority being exon skipping (SE) or alternative 3′-splice sites, with little change across timepoints (Figure 5). There is a 2–3% change in splicing events (differential AS (DAS)) in wounded pepper leaves. In these DAS events, an increase in SE, intron retention (RI), and mutually exclusive exons (MXE) with reduced splicing of 3′- or 5′-sites (A3SS and A5SS, respectively).

**Figure 6:**
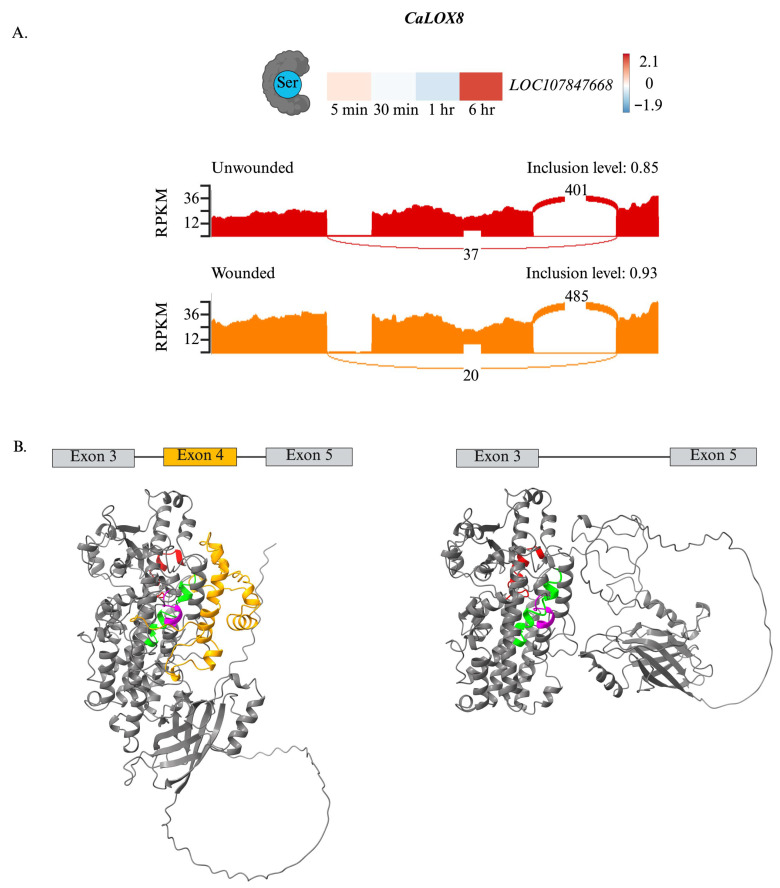
Alternative splicing of ***CaLOX8*** in wounded leaves may affect protein structure. At the four true leaf stage, individual pepper (*Capsicum annuum*) plants were either left undamaged (*n* = 4) or wounded (*n* = 4) on the second set of fully unfurled leaves using a hole-punch, removing approximately 12% of the leaf, avoiding the midvein. Foliar gene expression over a 6 h time course was measured by RNA-Seq (Supplemental Table S3A). Alternative splicing (AS) events were identified by rMATs. At 6 h post-wounding, an increased retention of exon 4 in ***CaLOX8*** in wounded leaves is predicted. (**A**) Top: Heatmap visualizing the frequency of ***CaLOX8*** AS events over the wounding time course. Bottom: Sashimi plot of splicing variants of ***CaLOX8*** in undamaged (top) and wounded (bottom) leaves at 6 h post-wounding. (**B**) Cartoon of ***CaLOX8*** splicing variants without (left) and with (right) Exon 4. In the models generated by importing AlphaFold3 structures into ChimeraX. The following color-coding in the protein model is used: exons 3 and 5 are gray, exon 4 is orange, the fatty acid substrate-binding domain in exon 5 is red, the phosphosite in exon 7 is magenta, and the oxygen-binding site in exon 8 is lime green. Figure created with BioRender (https://www.biorender.com/).

As with tea CsLOXs [45], CaLOXs also may undergo AS (Figure 6). Notably, a change in ***CaLOX8*** splicing is predicted 6 h post-wounding. In contrast to undamaged plants, there is an 8% predicted higher retention of exon 4, which is the one preceding the exon containing the substrate-binding domain (exon 5), in wounded pepper leaves. Through protein modeling, retention of this exon may affect protein structure, potentially shielding access of the substrate to the active site, negatively affecting enzyme activity (Figure 6B).

### 2.5. Wound-Induced Expression of Genes Encoding Proteins Involved in Jasmonate Signaling in Pepper Leaves

**Figure 7:**
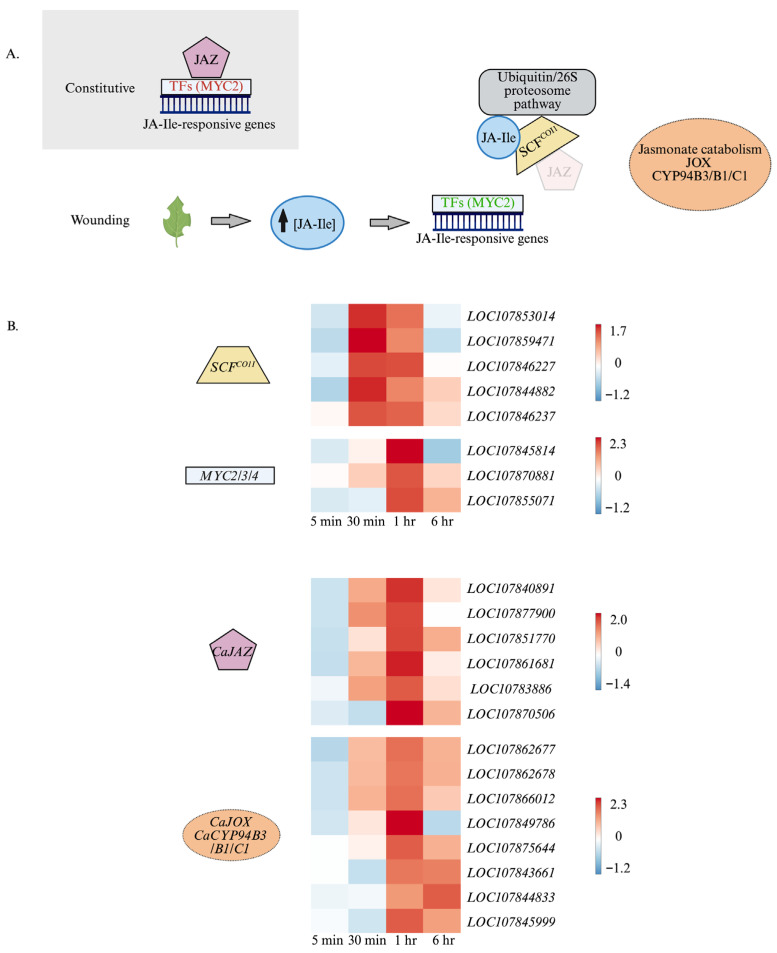
Expression of genes involved in the regulation of jasmonate signaling. At the four true leaf stage, individual pepper (*Capsicum annuum*) plants were either left undamaged (*n* = 4) or wounded (*n* = 4) on the second set of fully unfurled leaves using a hole-punch, removing approximately 12% of the leaf, avoiding the midvein. Foliar gene expression over a 6 h time course was measured by RNA-Seq, and wound-induced genes were identified by DESeq2 (Padj ≤ 0.05). (Supplemental Table S3A,B). (**A**) Model of the regulation of jasmonoyl-isoleucine (JA-Ile)-responsive gene expression. (**B**) Top: Heatmaps visualizing the expression of genes encoding positive regulators of JA-Ile signaling Bottom: heatmaps visualizing the expression of genes encoding negative regulators of JA-Ile signaling. Heatmaps represent the Z-score, with red indicating highly expressed genes. Abbreviations: jasmonate-induced oxidase (JOX); jasmonic acid-Zim domain proteins (JAZ), jasmonoyl-isoleucine (JA-Ile), transcription factors (TFs). Figure created with BioRender (https://www.biorender.com/).

As nuclear JA-Ile levels increase, JA-Ile bridges the SCF^COI1^ complex to JAZ proteins, resulting in their ubiquitination and degradation by the 26S-proteasome, releasing MCY2, MYC3, and MYC4 transcription factors from repression [33,34,35,36]. Within the first hour after wounding, transcript levels of components of the SCF^COI1^ complex increase, including those encoding F-box proteins (LOC107853014, LOC107859471) and E3-ubiquitin ligases (CaPUB22 (LOC107846227), CaPUB23 (LOC107844882, LOC107846237)). One hour after damage, wound-induced *CaMYC2* (*LOC107845814*, *LOC107870881*) and *CaMYC3* (*LOC107855071*) expression is observed (Figure 7A). A number of WRKY transcription factors, including *CaWRKY17* (*LOC107854937*), *CaWRKY22* (*LOC107839265*), *CaWRKY40* (*LOC107861978*, *LOC107876015*), and *CaWRKY53* (*LOC107839290*), are induced at early and mid-timepoints in wounded leaves.

### 2.6. Wound-Induced Expression of Genes Encoding Enzymes in Specialized Metabolism in Pepper Leaves: Non-Volatile and Volatile Compounds

Upregulation of transcripts encoding enzymes involved in non-volatile specialized metabolism was observed in pepper leaves 6 h after wounding. Wound-induced genes include enzymes involved in the biosynthesis of the tropane alkaloid calystegine (tropinone reductase (LOC107850005, LOC107867623)), triterpenoid and steroid biosynthesis (squalene epoxidase (LOC107840150)), β-amyrin synthase (LOC107841772), β-amyrin 28-monooxygenase (LOC107845824, LOC107859002), β-amyrin 11-oxidase (LOC107853307), and potentially involved in terpenoid indole alkaloid biosynthesis (7-deoxyloganetin glucosyltransferases (LOC107840056, LOC107867483) and stemmadenine O-acetyltransferase (LOC107873928)).

Early polyphenolic-related genes upregulated within the first hour after wounding include the transcription factor *CaMYB15* (*LOC107852599*), flavonoid biosynthesis-related genes (*trans-cinnamate: CoA ligase* (*LOC107859652*), *flavonol 4′sulfotransferase* (*LOC107845791*), *anthocyanidin 3-O-glucosyltransferase 2* (*LOC107860695*), and *agmatine hydroxycinnamoyl transferase 1* (*LOC107848097*)), and the lignin biosynthetic genes (*caffeoyl shikimate esterase* (*LOC107848284*), *caffeoyl-CoA O-methyltransferases* (*LOC107859652*, *LOC107860279*), and the *peroxidase* (*LOC17866097*) [49,50,51,52,53,54,55]. Later wound-induced transcript expression includes genes that encode enzymes in flavonoid biosynthesis (flavonoid 3′-hydroxylase CYP75B137 (LOC107867152, LOC107867141) and vestitone reductase (LOC107845154)) and lignin biosynthesis (peroxidases (LOC107840027, LOC107867619, LOC107842218, LOC107843551, LOC107860325, LOC107874093, LOC107863246, LOC107866096, LOC107866094, LOC107860938), dirigent protein 22 (LOC107849702), laccases (LOC107872619, LOC107874615)) [56,57,58].

A number of pepper genes that encode enzymes related to volatile biosynthesis are upregulated in response to wounding. Terpenoid genes are induced 1 h ((*R*)-*linalool synthase* (*LOC107840096*; monoterpenoid biosynthesis); (*3S,6E*)*-nerolidol synthase* (*LOC107845646*; sesquiterpenoid biosynthesis); *geranylgeranyl diphosphate synthase* (*LOC107856323*; diterpenoid biosynthesis)) and 6 h (*1-deoxy-D-xylulose-5-phosphate synthase 2* (*LOC107850768*), 2C-methyl-D-erythritol 4-phosphate pathway; *neomenthone dehydrogenase* (*LOC107839158*, monoterpenoid biosynthesis); (*3S,6E*)*-nerolidol synthase* (*LOC124892657*, *LOC107845646*; sesquiterpenoid biosynthesis))) after foliar wounding. As well, a downregulation of *eugenol synthase* (*LOC107870192*, monoterpenoid biosynthesis) is also observed at 6 h post-damage. The wound-induced upregulation of *acetyl-CoA benzyl alcohol acetyl transferases* (1 h: *LOC107866161*; 6 h: *LOC107879437*) may result in an increase in volatile benzyl ester emissions [59]. The wound-induced expression of *9-divinyl ether synthase* (30 min: *LOC107840369*) and *acetyl-CoA benzyl alcohol acetyl transferases* (1 h: *LOC107866161*; 6 h: *LOC107879437*) may result in an increase in volatile 9-divinyl ether and benzyl ester emissions [59,60].

### 2.7. Wound-Induced Expression of Classical Jasmonate Marker Genes in Pepper Leaves

Two groups of classic jasmonate-responsive gene markers [61,62,63], *proteinase inhibitors* (*LOC107866028*, *LOC107864939*, *LOC107862777*, *LOC107866027*, *LOC124897129*, *LOC124897130*, *LOC107843348*, *LOC107879783*, *LOC107879784*), and *defensins* (*LOC107852231*, *LOC124890736*, *LOC107877537*), are expressed late (6 h) after mechanical damage.

### 2.8. Wound-Induced Expression of Negative Regulators of Jasmonate Signaling

Pepper genes encoding negative regulators of jasmonate signaling are expressed as early as 30 min post-wounding. Genes encoding enzymes involved in jasmonate catabolism ultimately result in lower available JA-Ile. Cytochrome P_450_ enzymes B1/B3 and C1 are involved in the consecutive oxidation of JA-Ile to the 12-OH and 12-COOH, respectively [64,65,66]. *Cytochrome P_450_ B1/B3* genes (*LOC107862677*, *LOC107862678*, *LOC107866012*) increase at 30 min, peaking at 1 h and returning to lower but still wound-induced levels at 6 h, whereas the genes that encode cytochrome P_450_ 94C1 are induced at 1 h (LOC107849786; LOC107875644) or 6 h (LOC107843661) post-damage. Genes encoding CaJOX4 (LOC107845999) and CaJOX3 (LOC107844833), enzymes that oxidize jasmonic acid [67], are expressed 1 and 6 h after wounding, respectively (Figure 7B). A late-expressed gene encodes methyl jasmonate esterase 1 (LOC107840334) that catalyzes the conversion of methyl jasmonate to jasmonic acid [68]. Even if JA-Ile levels increase, this increase can be muted by JAZ repressors that bind to MYC2, MYC3, and MYC4 transcription factors [33,34,35]. Expression of most wound-induced *CaJAZ* genes begins as early as 30 min with highest levels at 1 h and returning to basal levels at 6 h post-wounding (*CaTIFY10A* (*LOC107840891*, *LOC107877900*), *CaTIFY10C* (*LOC107861681*), *CaTIFY5A* (*LOC107838868*)), with the exception being *CaTIFY10B* (*LOC107851770*) and *CaJAZ7* (*LOC107870506*) that are only wound-induced at 1 h [69] (Figure 7B).

In arabidopsis, splice variants of JAZ desensitize the plant to jasmonate signaling [46,47]. These variants lack the C-terminal jas motif; therefore, they can bind and repress the MYC transcription factors but cannot be targeted for degradation, and, thus, repression of jasmonate signaling is maintained regardless of high JA-Ile concentration. In wounded pepper leaves, over this time course, we did not observe alternative splicing of JAZ genes over the time course of our experiment.

## 3. Discussion

**Figure 8:**
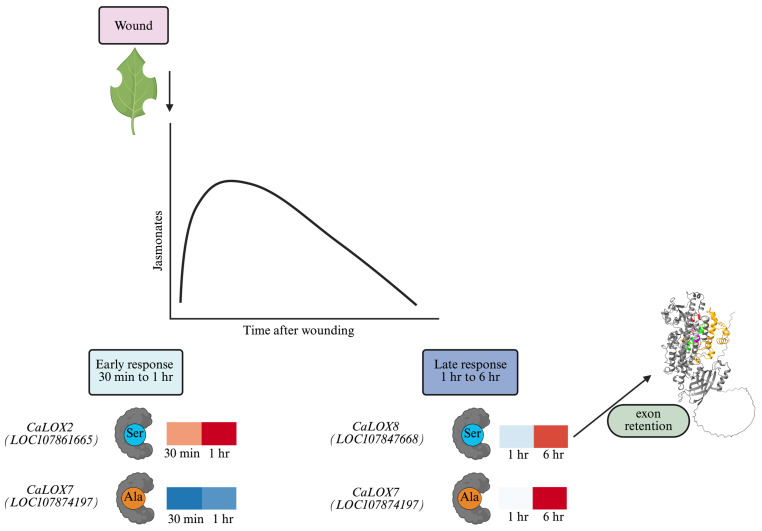
Model of dynamic jasmonate signaling in wounded *Capsicum annuum* leaves. The dynamic changes in jasmonoyl-isoleucine (JA-Ile) levels reflect the transcription and post-translational regulation of the genes and proteins involved in jasmonate biosynthesis and signaling. JA-Ile levels increase within the first few minutes after wounding, reaching maximal levels after 1 h and decreasing by 6 h. In the early response to wounding (30 min, 1 h), there is an increase in the expression of genes encoding jasmonate biosynthetic enzymes (CaPLA, CaLOX, CaAOS, CaAOC, CaOPR3, CaAIM1) (Figure 3). Further positive regulation of the jasmonate pathway is observed with the upregulation of *F-box proteins* and the *E3 ubiquitin ligases PUB22/23,* as well as the *MYC2/3/4* transcription factors (Figure 7B). Specifically focusing on CaLOXs, ***CaLOX8*** and ***CaLOX2***, which are rapidly expressed in response to wounding, both encode proteins with a Ser at the predicted phosphosite that may be dephosphorylated in response to wounding, contributing to the initial wound-associated jasmonate burst. At 6 h post-wounding, increased retention of exon 4 of ***CaLOX8*** is predicted to shield the enzymes’ active site, resulting in lower ***CaLOX8*** activity. At this timepoint, ***CaLOX7***, which encodes a protein with an Ala at the phosphosite, is expressed, which may contribute to sustained jasmonate production. Increased expression of *JAZ* genes is also observed, tempering jasmonate signaling (Figure 7B). Jasmonate signaling is shut off in the late response through the induction of genes encoding the jasmonate-deactivating jasmonate-induced oxygenases (JOXs) and members of the cytochrome P_450_ 94 family (CYP94). Heatmaps represent the ratio of gene expression in wounded/undamaged plants, with red indicating high gene expression. Figure created with BioRender (https://www.biorender.com/).

In response to foliar wounding, pepper JA and JA-Ile levels increased rapidly to peak around 1 h post-damage and then returned to basal levels (Figure 1). This dynamic fluctuation reflects the regulation of jasmonate biosynthesis and jasmonate signaling. In this study, we focused on understanding the potential role of transcription, alternative splicing, and, indirectly, post-translational phosphorylation through the presence of a phosphosite on the regulation of pepper 13*S*-LOXs that catalyze an early step in jasmonate biosynthesis (Figure 8).

### 3.1. Jasmonate Profile Reflects Genes Involved in Jasmonate Biosynthesis, Signaling and Repression

In general, a strong upregulation of genes that encode jasmonate biosynthetic enzymes, including phospholipases, **CaLOX2**, **CaLOX7**, CaAOS1, CaAOS3, CaAOC, CaOPR3, and CaAIM, is observed (Figure 3 and Figure 4). In arabidopsis, an enzyme early in jasmonate biosynthesis, AtLOX2, has been identified as being involved in the regulation of flux through this pathway. This enzyme is constitutively phosphorylated at Ser^600^ and dephosphorylated in response to wounding [41]. In vitro studies with phosphovariants show that AtLOX2 phosphomimics have basal activity, which increases 10-fold in the phosphonull mutants [42]. Together, this supports a model where enzymes for jasmonate biosynthesis are constitutively present and flux through the pathway reflects substrate availability and post-translational regulation, such as the phosphorylation of AtLOX2 [10,42]. Pepper has four 13*S*-LOXs that may be involved in jasmonate biosynthesis [43]. Of interest, some of these CaLOXs, **CaLOX2** and **CaLOX8**, have a Ser at their homologous phosphosite, suggesting that they may be regulated through phosphorylation, whereas other CaLOXs, **CaLOX6** and **CaLOX7**, have a non-phosphorylatable Ala at this site. We hypothesize that **CaLOX2** and **CaLOX8** are constitutively expressed and involved in early stress-associated jasmonate biosynthesis compared to **CaLOX6** and **CaLOX7**, which may be involved in longer, more sustained jasmonate production. In support of this, ***CaLOX2***, which encodes an enzyme with a Ser at the phosphosite, is expressed early after wounding, in contrast to ***CaLOX7***, which encodes an enzyme with an Ala at the phosphosite, that is highly expressed later in the time course (Figure 4). A similar pattern was observed in a previous study by Sarde et al. [70] in response to thrip, *Frankliniella occidentalis*, infestation or JA application to pepper leaves; they observed early induced expression of ***CaLOX2*** and the later strong expression of ***CaLOX7***. They observed expression of ***CaLOX6*** at 8- and 24 h post-JA treatment, which was longer than the time course conducted in our experiment. Ongoing studies generating transgenic plants with phosphovariants are being conducted to fully understand the role of LOX phosphophorylation at this Ser in arabidopsis and pepper.

In addition to transcriptional and post-translational regulation, genes are also regulated through AS [44]. At each timepoint, approximately 350,000 AS events were identified, with a 2–3% difference in AS events between unwounded and wounded pepper leaves. The dominant types of differential AS events were SE, MXE, and RI, which are consistent with a meta-analysis of stress-related splicing events in pepper [71] (Figure 5).

***CaLOX8*** is constitutively expressed in pepper leaves and not wound-induced (Figure 4); however, a differential AS exon skipping event was identified for this transcript. In wounded leaves at 6 h, there is a predicted increase in the retention of the 4th exon of ***CaLOX8***, which is the exon preceding exon 5 that contains the substrate-binding domain (Figure 6). Modeling with AlphaFold3 suggests that this may block fatty acid substrate access to the active site, potentially negatively affecting enzyme activity, but this must be confirmed experimentally through enzyme assays with recombinant isozymes and validation of the different isozymes *in planta* (Figure 6B).

In support of jasmonate signaling, *CaMYC2* (*LOC107845814*, *LOC107870881*), *CaMYC3* (*LOC107855071*), and components of the CaSCF^COI1^ complex (E3-ubiquitin ligases (CaPUB22 (LOC107846227), CaPUB23 (LOC107844882, LOC107846237); F-box proteins (LOC107853014, LOC107859471)) are upregulated in wounded pepper leaves (Figure 7A) [33,34,35]. Also, genes encoding a number of CaWRKY transcription factors known to be involved in jasmonate signaling are wound-induced. AtWRKY17 is involved in jasmonate × salicylic acid (SA) crosstalk and decreases SA signaling by positively regulating *Pseudomonas syringae*-induced jasmonate accumulation in arabidopsis [72,73]. AtWRKY40 supports jasmonate signaling by repressing the expression of SA-responsive genes as well as JAZ repressors during infection by the powdery mildew fungus *Golovinomyces orontii* [74]. AtWRKY53 leads to the expression of *AtWRKY22* that negatively affects both SA- and jasmonate-responsive gene expression [75]. Of interest, *CaWRKY53* (*LOC107839290*) is highly expressed at 30 min post-wounding. In arabidopsis, AtWRKY53 interferes with jasmonate signaling by negatively regulating *AtLOX3* and *AtLOX4* expression (Jiao et al., 2022) [76].

Contributing to the dynamic nature of jasmonate signaling, expression of genes that encode proteins that attenuate jasmonate-signaling is observed at 6 h post-wounding (Figure 2 and Figure 7B). In arabidopsis, cytochrome P_450_ 94B1/B3 catalyzes the oxidation of JA-Ile to 12OH-JA-Ile [64,66]. This is followed by the action of cytochrome P_450_ 94C1 that further oxidizes 12OH-JA-Ile to 12COOH-JA-Ile. Pepper *cytochrome P_450_ 94B1/B3* genes are expressed early, peaking at 1 h post-wounding, compared to *cytochrome P_450_ 94C1*. In arabidopsis, *AtCYP94C1* was also induced by wounding and treatment with methyl jasmonate [77].

Jasmonate oxidases (JOXs) catalyze the oxidation of jasmonic acid, removing its availability to be conjugated to isoleucine by JAR1 [31,67,78]. Pepper *CaJOX4* and *CaJOX3* genes are wound-induced at 1 h, with *CaJOX3* expression increasing at 6 h after damage (Figure 7B). *Methyl jasmonate esterase I* (*LOC107840334*) is induced 6 h post-wounding and encodes an enzyme that catalyzes the demethylation of methyl jasmonate to jasmonic acid [68]. The expression of genes that encode JAZ proteins, which bind to MYC2, MYC4, and MYC6 transcription factors and repress jasmonate-responsive gene expression, is observed within 30 to 60 min of wounding and decreases to basal levels at 6 h (Figure 7B). Expression of *CaJAZ* genes was also observed when leaves were treated with jasmonic acid [79]. In arabidopsis, alternative splicing of JAZ transcripts produces proteins without a C-terminus jas motif, thereby reducing their degradation when JA-Ile levels are high and contributing to sustained negative regulation of jasmonate signaling [46,47]. In wounded pepper leaves, we did not observe alternative splicing of JAZ genes over the time course of our experiment.

### 3.2. Dynamic Jasmonate Levels Are Reflected in Specialized Metabolism Gene Expression

The fluctuations of JA-Ile levels in wounded pepper leaves translate into transcriptional reprogramming and upregulation of genes involved in specialized metabolism. Genes that encoded enzymes involved in non-volatile specialized metabolism biosynthesis were induced in pepper leaves 6 h post-damage. These genes encode enzymes in the general triterpenoid/steroidal pathway, including squalene epoxidase (LOC107840150), which catalyzes the oxidation of squalene to 2,3-oxidosqualene, and β-amyrin synthase (LOC107841772), which converts 2,3-oxidosqualene to β-amyrin [80,81]. In *Glycyrrhiza glabra* (licorice), β-amyrin is oxidized to 11-*oxo*-β-amyrin through the action of β-amyrin 11-oxidase [82]. This precursor can take the path to oleanane-type triterpenoid saponins through the action of β-amyrin 2,8-monooxygenase (LOC107845824, LOC107859002, LOC107859002) [80].

Even though pepper is not thought to produce terpenoid indole alkaloids, *7-deoxyloganetin glucosyltransferases* (*GGTs*) (*LOC107840056*, *LOC107867483*) and *stemmadenine O-acetyltransferase* (*LOC107873928*) were wound-induced. In the Madagascar periwinkle, *Catharanthus roseus*, 7-deoxyloganetin glucosyltransferase catalyzes the conversion of the iridoid 7-deoxyloganetic acid to 7-deoxyloganic acid, which is enzymatically converted to secologanin through a number of steps [83]. A number of *CaGGT* genes are known to be encoded in the *C. annuum* genome [84]. In *C. roseus*, later in the biosynthetic pathway leading to the indole alkaloid catharanthine, stemmadenine O-acetyltransferase catalyzes the acetylation of stemmadine to stemmadenine acetate [85]. The presence of related enzymes in peppers suggests that similar indole-type alkaloids may be present in pepper leaves.

Tropinone reductase (LOC107850005, LOC107867623) reduces tropinone to pseudotropine in the biosynthesis of the insecticidal tropane alkaloids calystegine [86,87].

Phenylpropanoid compounds are important defensive specialized metabolites, including lignins that are part of structural barriers and flavonoids that confer resistance against insect pests and pathogens [88,89]. Early expression of the gene that encodes the transcription factor CaMYB15 (LOC107852599) positively regulates the expression of genes involved in phenylpropanoid biosynthesis [90,91]. The lignin biosynthesis-related biosynthetic genes *caffeoyl shikimate esterase* (*LOC107848284*) and *caffeoyl-CoA O-methyltransferase* (*LOC107859652*, *LOC107860279*) were expressed early after wounding, whereas genes encoding peroxidases, laccases, and dirigent proteins that may be involved in lignin biosynthesis are expressed at 6 h after wounding [49,50,53,56,58,92,93,94]. Flavonoid genes upregulated in response to wounding included *flavonol 4′sulfotransferase* (*LOC107845791*), *flavonoid 3′-hydroxylase* (*LOC107867152*, *LOC107867141*), and *anthocyanidin 3-O-glucosyltransferase 2* (*LOC107860695*) [51,54,55,94,95]. Strong upregulation of the genes encoding 2-hydroxyisoflavanone dehydratase (LOC107856532) and vestitone reductase (LOC107856532) is noted 6 h post-wounding. In legumes, 2-hydroxyisoflavanone dehydratase catalyzes the conversion of flavonones to isoflavones. Farther down the isoflavonoid pathway, vestitone reductase catalyzes the NADPH-dependent reduction in (3R)-vestitone to 7,2′-dihydroxy-4′-methyoxylisoflavanol that then can be enzymatically converted into phytoalexins such as medicarpin, vestitol, and sativan [96,97,98,99]. The gene encoding vestitone reductase is strongly upregulated in the roots of resistant pepper infected with the oomycete *Phytophthora capsica* [57]. The gene encoding agmatine hydroxycinnamoyl transferase 1 (LOC107848097, OC107846882), which is involved in hydroxycinnamic acid aldehyde biosynthesis, is induced in wounded pepper leaves. In many plant species, agmatine hydroxycinnamic acid amides have been shown to play a role in plant defense against pathogens and insect herbivores [100,101,102,103,104,105].

In response to foliar damage, the production and release of plant volatile compounds may convey the presence of insect herbivores to natural enemies [106,107]. Twenty-four hours after infestation by caterpillars of the beet armyworm, *Spodoptera exigua*, pepper volatile emissions increase 7-fold, and these volatiles include the monoterpene linalool and the sesquiterpene nerolidol [108]. Linalool was also a primary leaf volatile emitted in response to *Tetranchus urticae*, two-spotted spider mite, infestation [79]. In our study, genes, in general, terpenoid pathways (*1-deoxy-D-xylulose-5-phosphate synthase 2* (*LOC107850768*), 2C-methyl-D-erythritol 4-phosphate pathway), as well as later terpenoid biosynthetic enzymes ((*R*)-*linalool synthase* (*LOC107840096*; monoterpenoid biosynthesis), (*3S,6E*)*-nerolidol synthase* (*LOC107845646*, *LOC124892657*; sesquiterpenoid biosynthesis) that are involved in linalool and nerolidol biosynthesis are wound-induced. Six hours after foliar damage, expression of *neomenthone dehydrogenase* (*LOC107839158*, monoterpenoid biosynthesis), which catalyzes menthone biosynthesis, is observed, which may be involved in resistance to pathogens that potentially could invade the plant through the leaf wound [109]. *Divinyl ether synthase* genes are upregulated in response to infection of pepper leaves with the Obuda pepper virus (Tobamovirus) [110]. In tobacco, the divinyl ethers colneleic acid and colnelenic acid exhibit potent antimicrobial effects on the oomycete *Phytophthora parasitica* var. nicotianae [111].

In the late response after wounding, classic jasmonate-responsive genes encoding proteinase inhibitors (LOC107866028, LOC107864939, LOC10782777, LOC107866027, LOC124897129, LOC124879130, LOC107843348, LOC107879783, LOC107879784) and defensins (LOC107852231, LOC124890736, LOC107877537) are strongly upregulated [61,62,63].

### 3.3. Conclusions

In response to foliar wounding, jasmonate levels regulate plant defense responses. Therefore, understanding their regulation is key to enhancing plant resistance to pathogens and chewing insect herbivores. The dynamic levels of JA and JA-Ile reflect the transcriptional, alternative splicing, and post-translational regulation of CaLOXs (Figure 1, Figure 3, Figure 4, and Figure 8). All *CaLOXs* are constitutively expressed. In response to damage, **CaLOX2**, which has a Ser in its regulatory phosphosite, is expressed early after wounding of pepper leaves, supporting the possibility that this protein is regulated by phosphorylation and, in response to wound stress, a phosphatase is activated to dephosphorylate these enzymes, increasing flux into jasmonate biosynthesis. In contrast, wound-induced ***CaLOX7***, which encodes an enzyme with Ala at the predicted phosphosite, is highly expressed at a later timepoint (6 h after wounding) that potentially could contribute to a more long-term sustained jasmonate biosynthesis. The alternative splicing pattern of ***CaLOX8*** may also be affected by wounding; 6 h post-wounding, an 8% increase in the retention of exon 4 is predicted. Modeling suggests that retention of exon 4 may block access of the active site to the substrate, negatively affecting jasmonate biosynthesis (Figure 6). The rapid increase in JA and JA-Ile leads to the expression of genes encoding jasmonate biosynthetic and signaling proteins, including CaPLA1, **CaLOX2**, CaAOS, CaAOC, CaOPR2, and CaMYC2, 1 h post-wounding (Figure 3 and Figure 7A). At 6 h post-stress, expression of genes encoding JOX enzymes, which convert JA to the inactive 12-OH-JA, and JAZ repressors is observed, which contributes to the lower levels of JA and JA-Ile and wound-associated gene expression observed (Figure 1, Figure 2, and Figure 7B). Given the importance of jasmonates in plant resistance to necrotrophic pathogens and chewing insect herbivores [4,5,6], understanding the regulation of jasmonate biosynthesis is key to enhancing plant protection. These results clarify the possible regulation of LOXs at the transcriptional, splicing, and post-translational levels that lead to the dynamic, stress-associated jasmonate profiles.

## 4. Materials and Methods

### 4.1. Plant Maintenance

Three seeds (*Capsicum annuum* ’Mini Bell’ source: Richters (Goodwood, ON, Canada) were sown per pot (15.2 cm × 11.4 cm, d × h) containing Fafard Agro G6 potting mix (Scotts, Saint-Bonaventure, QC, Canada) and placed in a Conviron growth cabinet (Winnepeg, MB, Canada) at conditions set to emulate the average summer conditions in Montréal, Québec (day: 14 h at 25 °C; ramped to night over 2.5 h; night: 5 h at 22 °C, ramped to day over 2.5 h) with a light intensity of 250 µmoles/m^2^/s. At two weeks, seedlings were thinned to a single plant per pot. Four-week-old plants were used in experiments.

### 4.2. Wounding Experiment

Once the pepper plants had two sets of fully expanded leaves (~four weeks), a wounding time course was performed. Three days before the experiment, plastic shields were used to separate plant treatments (undamaged vs. damaged) to prevent volatile signaling. At noon, half of the plants were wounded multiple times using a hole punch to damage approximately 12% of the second set of fully unfurled leaves from the apex, avoiding the midvein. Damaged leaves from wounded plants or comparable leaves from undamaged plants were taken at 5 min, 30 min, 1 h, and 6 h after wounding and immediately frozen in liquid nitrogen and stored at −80 °C. These timepoints represent early and late times after wounding. Plants were not resampled; once leaves were removed, the plant was discarded. At each timepoint, leaves were taken for phytohormone and transcriptomic analyses. As well, unwounded and damaged plants were used to determine tissue losses due to wounding by comparing the dry weight (DW) of wounded and unwounded above-ground portions (Supplemental Table S4). The experiment was temporally repeated 4 times.

### 4.3. Jasmonate Quantification

Acidic jasmonates (OPDA, JA, JA-Ile) quantification was conducted based on Glauser et al. [112]. Pepper leaves were lyophilized, homogenized, and extracted in HPLC-grade methanol:water (70:30) containing a mixture of isotopically-labeled phytohormone internal standards (D6-JA and D6-JA-Ile (HPC Standards)). Following homogenization (Retsch Mill MM 400 (Düsseldorf, Germany)), 10 min, vibration 30 Hz), extracts were centrifuged (18,994× *g*, 20 min, 20 °C), and the supernatant was transferred to a new tube and evaporated to dryness in a speed-vacuum (Labconco, Kansas City, MI, USA) at room temperature. Using sonication, the pellet was resuspended in methanol/water (70:30) and recentrifuged (18,994× *g*, 5 min, room temperature) before separation by ultrahigh performance liquid chromatography (UPLC, Waters Acquity, Millford, MA, USA) coupled to a mass spectrometer (MS) (Bruker Elite EvoQ triple-quadrupole, Billerica, MA, USA).

Compounds were separated by UPLC chromatography on a Zorbax Eclipse XDB-C18 column (4.6 × 50 mm, 1.8 μm, Agilent, Santa Clara, MA, USA) maintained at a temperature of 42 °C. The mobile phase went from (A) 5% ACN, 0.1% formic acid to (B) 50% ACN, 0.1% formic acid to (C) 100% ACN, 0.1% formic acid using the following gradient: 30 s. of A, 10 s gradient of A to B, 90 s from B to C, where it was held for 1 min before returning to initial conditions over the next min. All solvents used were LC-MS grade. The flow rate was 400 μL/min, and the column was maintained at a temperature of 42 °C.

After separation, compounds were nebulized by electron spray ionization (negative mode) under the following conditions: capillary voltage 4500, cone 35 arbitrary units (a.u.)/350 °C, probe 60 a.u./475 °C, and nebulizer gas (N_2_) 60 a.u. Data processing was conducted using MS Data Review software (Bruker MS Workstation, vers. 8.2).

Phytohormones were identified based on retention time and the transition *m*/*z* (Supplemental Table S5). Foliar phytohormone levels were calculated based on the peak area of the compound-of-interest normalized to the corresponding internal standard divided by the initial dry weight of leaf material.

Four samples were taken per treatment at each timepoint. However, in undamaged samples, JA levels are extremely low and occasionally below detection limits. Thus, a one-way analysis-of-variance (ANOVA) (factor: time) on wounded samples was performed with SPSS (version 29.0.2.0) software. Significant differences were determined by the Tukey honestly significant difference (HSD) *post hoc* test.

### 4.4. Transcriptomics: RNA-Seq

Plant tissue (~50 mg) was finely ground in liquid nitrogen using a sterile mortar and pestle and transferred to a pre-cooled Eppendorf tube. Total RNA was extracted using the RNeasy Plant Mini Kit (Qiagen (Hilden, Germany)). Once the liquid nitrogen had evaporated, guanidine thiocyanate (RLT) lysis buffer was added to the tissue and vortexed. The lysate was transferred into a QIAshredder column and centrifuged for two minutes (12,000× *g*). Ethanol (0.5 volume) was added, and the ethanol/lysate solution was centrifuged (10,000× *g*) for 45 s in an RNeasy mini spin column in a collection tube. After discarding the flow-through, the column was washed with RW1 and centrifuged (10,000× *g*, 45 s), this was followed by washing with RPE and centrifugation (10,000× *g*, 45 s and then 150 s). After each step, the flow-through was discarded. The RNeasy spin column was transferred into a new collection tube, and 30 µL of RNAse-free water was applied directly to the column’s membrane. A final 1 min centrifugation (≥8000× *g*) eluted the RNA.

RNA concentration and sample purity were determined using a Nanodrop spectrophotometer (ThermoFisher Scientific (Waltham, MA, USA)). To further confirm the quality of the RNA sample, it was separated on a bleach gel [113]. Total RNA was sent for library preparation and next-generation sequencing (100 paired-end reads) at McGill University and Genome Quebec Innovation Centre using the Illumina NovaSeq 6000 platform (San Diego, CA, USA). A polyA-enriched RNA library was used, and a consistent sequencing depth of ~100,000,000 reads was obtained. The Digital Research Alliance of Canada’s Narval Unix-based server was used to store data and to perform computations. After removal of the adaptor and quality control using FastQC [114], reads were aligned to the *C. annuum* reference genome UCD10Xv1.1 obtained from the National Center for Biotechnology Information (NCBI) using STAR version 2.7.11b [115] (Supplemental Tables S2 and S3A). The raw read data (FASTQ) from this study have been deposited in the NCBI Sequence Read Archive (Bioproject ID #PRJNA1348599).

Differential expression analysis and visualization (i.e., volcano, ridgeline, and heatmap plots) were performed with DESeq2 within the ExpressAnalyst platform [116,117]. DESeq2 was applied to identify wound-induced genes at each timepoint. Differentially expressed genes (DEGs) were filtered with a log fold-change (LogFC) cutoff of above 0.95 or below −0.95 and an adjusted *p*-value ≤ 0.05 (Supplemental Table S3B).

The expression pattern of DEGs across timepoints (5, 30, 60, and 360 min) was clustered using a smoothing spline clustering framework [118,119]. The raw count data of DEGs were normalized through DESeq2 variance stabilizing transformation [116]. For each gene, fitted cubic smoothing splines were subsequently standardized to z-scores to emphasize patterns over absolute expression. Candidate cluster numbers were evaluated using k-means, and the optimal k was selected according to the maximum average silhouette width.

To identify alternative splicing (AS) events and determine if they are differentially expressed across treatments, the rMATs tool was applied to the alignment files generated by STAR using the NovelSS feature [120]. To increase detection of novel splice junctions, the two-pass mode was applied, where the junctions detected in the first mapping pass are used as annotated junctions in the second pass. The software parameters were set to provide the alignments in both genomic and transcript coordinates, as well as quantification of the number of reads per gene. At each timepoint, wounded samples were compared to unwounded samples to identify differential AS events. The rMATS output was run through the rMATS to sashimiplot tool (version 3.0.0) to visualize differential AS events as sashimi plots [120]. To compare the **CaLOX8** protein structure with or without exon 4, AlphaFold3 models were overlaid in ChimeraX [121,122].

### 4.5. Pepper Lipoxygenases

The pepper Ca13*S*-LOXs used in this study (**CaLOX2** (LOC107861665), **CaLOX6** (LOC107874182), **CaLOX7** (LOC107874197), and **CaLOX8** (LOC107847668)) follow the nomenclature of Sarde et al. [43], who identified these proteins through comparative proteomics, ensuring they had signature lipoxygenase and PLAT/LH2 domains and phylogenetic analyses. **CaLOX2** (CAN.G862.55), **CaLOX7** (CAN.G649.19), and **CaLOX8** (CAN.G1931.1) were also identified as being 13*S*-lipoxygenases by Oliviera Camargo et al. [123]. **CaLOX6** (partial sequence 13-LOXc) and **CaLOX7** (13-LOXa) were identified as 13*S*-lipoxygenases by Juhasz et al. [124]. Throughout this paper, pepper CaLOXs have been color-coded; those with an **Ala** at the phosphosite (**CaLOX6** and **CaLOX7**) are in **orange**, and those with a **Ser** (**CaLOX2** and **CaLOX8**) are in **blue**.

## Data Availability

The transcriptomic data generated by this study are available in the Supplementary Materials of this article (Supplemental Table S3), and the raw read data (FASTQ) have been deposited in the NCBI Sequence Read Archive (Bioproject ID # PRJNA1348599).

## Supplementary Materials

The following supporting information can be downloaded at: https://www.mdpi.com/article/10.3390/plants15010045/s1. Supplemental Figure S1: Gene expression in wounded pepper (*Capsicum annuum*) leaves; Supplemental Table S1: Phytohormone statistics; Supplemental Table S2: RNA-Seq read summary; Supplemental Table S3A: Pepper (*Capsicum annuum*) transcriptomic data; Supplemental Table S3B: Pepper (*Capsicum annuum*) wound-induced differentially expressed genes (DEGs); Supplemental Table S4: Biomass loss due to mechanical wounding; Supplemental Table S5. Multiple reaction monitoring mass-to-charge ratios.

## Abbreviations

The following abbreviations are used in this manuscript:

A3SS	Alternative 3′-splicing site
A5SS	Alternative 5′ splicing site
ACN	Acetonitrile
ACX	Acyl-CoA oxidase
ANOVA	Analysis-of-variance
AOC	Allene oxide cyclase
AOS	Allene oxide synthase
AS	Alternative splicing
AUs	Arbitrary units
DAS	Differential alternative splicing
DEG	Differentially expressed gene
HSD	Honestly significant difference
JA	Jasmonic acid
JA-Ile	Jasmonoyl-isoleucine
JAR1	Jasmonate-resistant 1
JAZ	Jasmonate Zim-domain
(12*S*-13*S*)-EOT	(12*S*-13*S*)-Epoxy-hydroperoxy-octadecatrienoic acid
13-HPOT	(13*S*)-Hydroperoxy-octadecatrienoic acid
KAT	3-Ketoacyl CoA thiolase
MFP	Multifunctional protein
MXE	Mutually exclusive exons
OPDA	(9*S*,13*S*)-12-*oxo*-phytodienoic acid
OPR	OPDA reductase
RI	Intron retention
SCF^COI1^	Skp1/Cullin1/F-box protein coronatine-insensitive 1
SE	Skipped exon
SSP	Smoothing spline plot
UPLC-MS	Ultrahigh-performance liquid chromatography–mass spectrometry

## References

[1] Yuan Z., Zhang D. (2015). Roles of jasmonate signalling in plant inflorescence and flower development. Curr. Opin. Plant Biol..

[2] Huang H., Liu B., Liu L., Song S. (2017). Jasmonate action in plant growth and development. J. Exp. Bot..

[3] Zhao X., Li N., Song Q., Li X., Meng H., Luo K. (2021). OPDAT1, a plastid envelope protein involved in 12-oxo-phytodienoic acid export for jasmonic acid biosynthesis in *Populus*. Tree Physiol..

[4] Koo A.J. (2018). Metabolism of the plant hormone jasmonate: A sentinel for tissue damage and master regulator of stress response. Phytochem. Rev..

[5] Howe G.A., Major I.T., Koo A.J. (2018). Modularity in jasmonate signaling for multistress resilience. Annu. Rev. Plant Biol..

[6] Li C., Xu M., Cai X., Han Z., Si J., Chen D. (2022). Jasmonate signaling pathway modulates plant defense, growth, and their trade-offs. Int. J. Mol. Sci..

[7] Gfeller A., Baerenfaller K., Loscos J., Chételat A., Baginsky S., Farmer E.E. (2011). Jasmonate controls polypeptide patterning in undamaged tissue in wounded *Arabidopsis* leaves. Plant Physiol..

[8] Kaur D., Schedl A., Lafleur C., Martinez Henao J., van Dam N.M., Rivoal J., Bede J.C. (2024). Arabidopsis transcriptomics reveals the role of lipoxygenase2 (AtLOX2) in wound-induced responses. Int. J. Mol. Sci..

[9] Wasternack C., Hause B. (2013). Jasmonates: Biosynthesis, perception, signal transduction and action in plant stress response, growth and development. An update to the 2007 review in Annals of Botany. Ann. Bot..

[10] Kimberlin A.N., Holtsclaw R., Zhang T., Mulaudzi T., Koo A.J. (2022). On the initiation of jasmonate biosynthesis in wounded leaves. Plant Physiol..

[11] Bannenberg G., Martínez M., Hamberg M., Castresana C. (2009). Diversity of the enzymatic activity of the lipoxygenase gene family of *Arabidopsis thaliana*. Lipids.

[12] Farmer E.E., Goossens A. (2019). Jasmonates: What allene oxide synthase does for plants. J. Exp. Bot..

[13] Stenzel I., Hause B., Miersch O., Kurz T., Maucher H., Weichert H., Ziegler J., Feussner I., Wasternack C. (2003). Jasmonate biosynthesis and the allene oxide cyclase family of *Arabidopsis thaliana*. Plant Mol. Biol..

[14] Taki N., Sasaki-Sekimoto Y., Obayashi T., Kikuta A., Kobayashi K., Ainai T.P., Yagi K., Sakurai N., Suzuki H., Masuda T. (2005). 12-Oxo-phytodienoic acid triggers expression of a distinct set of genes and plays a role in wound-induced gene expression in Arabidopsis. Plant Physiol..

[15] Dave A., Graham I.A. (2012). Oxylipin signaling: A distinct role for the jasmonic acid precursor *cis*-(+)-12-oxo-phytodienoic acid (*cis*-OPDA). Front. Plant Sci..

[16] Theodoulou F.L., Job K., Slocombe S.P., Footitt S., Holdsworth M., Baker A., Larson T.R., Graham I.A. (2005). Jasmonic acid levels are reduced in COMATOSE ATP-binding cassette transporter mutants. Implications for transport of jasmonate precursors into peroxisomes. Plant Physiol..

[17] Guan L., Denkert N., Eisa A., Lehmann M., Sjuts I., Weiberg A., Soll J., Meinecke M., Schwenkert S. (2019). JASSY, a chloroplast outer membrane protein required for jasmonate biosynthesis. Proc. Natl. Acad. Sci. USA.

[18] Schaller F., Biesgen C., Mussig C., Altmann T., Weiler E.W. (2000). 12-Oxophytodienoate reductase 3 (OPR3) is the isoenzyme involved in jasmonate biosynthesis. Planta.

[19] Stintzi A., Browse J. (2000). The *Arabidopsis* male-sterile mutant, *opr3*, lacks the 12-oxophytodienoic acid reductase required for jasmonate synthesis. Proc. Natl. Acad. Sci. USA.

[20] Strassner J., Schaller F., Frick U.B., Howe G.A., Weiler E.W., Amrhein N., Macheroux P., Schaller A. (2002). Characterization and cDNA-microarray expression analysis of 12-oxophytodienoate reductases reveals differential roles for octadecanoid biosynthesis in local versus the systemic wound response. Plant J..

[21] Koo A.J.K., Chung H.S., Kobayashi Y., Howe G.A. (2006). Identification of a peroxisomal acyl-activating enzyme involved in the biosynthesis of jasmonic acid in Arabidopsis. J. Biol. Chem..

[22] Cruz Castillo M., Martínez C., Buchala A., Métraux J.-P., Léon J. (2004). Gene-specific involvement of β-oxidation in wound-activated responses in Arabidopsis. Plant Physiol..

[23] Afitlhile M.M., Fukushige H., Nishimura N., Hildebrand D.F. (2005). A defect in glyoxysomal fatty acid β-oxidation reduces jasmonic acid accumulation in *Arabidopsis*. Plant Physiol. Biochem..

[24] Pinfield-Wells H., Rylott E.L., Gilday A.D., Graham S., Job K., Larson T.R., Graham I.A. (2005). Sucrose rescues seedling establishment but not germination of Arabidopsis mutants disrupted in peroxisomal fatty acid catabolism. Plant J..

[25] Delker C., Zolman B.K., Miersch O., Wasternack C. (2007). Jasmonate biosynthesis in *Arabidopsis thaliana* requires peroxisomal β-oxidation enzymes—Additional proof by properties of pex6 and aim1. Phytochemistry.

[26] Schilmiller A.L., Koo A.J., Howe G.A. (2007). Functional diversification of acyl-coenzyme A oxidases in jasmonic acid biosynthesis and action. Plant Physiol..

[27] Li M., Yu G., Cao C., Liu P. (2021). Metabolism, signaling, and transport of jasmonates. Plant Commun..

[28] Ishimaru Y., Oikawa T., Suzuki T., Takeishi S., Matsuura H., Takahashi K., Hamamoto S., Uozumi N., Shimzu T., Seo M. (2017). GTR1 is a jasmonic acid and jasmonoyl-L-isoleucine transporter in *Arabidopsis thaliana*. Biosci. Biotechnol. Biochem..

[29] Nguyen C.T., Martinoia E., Farmer E.E. (2017). Emerging jasmonate transporters. Mol. Plant.

[30] An N., Huang X., Yang Z., Zhang M., Ma M., Yu F., Jiang L., Du B., Wang Y.-F., Zhang X. (2024). Cryo-EM structure and molecular mechanism of the jasmonic acid transporter ABCG16. Nat. Plants.

[31] Staswick P.E., Tiryaki I. (2004). The oxylipin signal jasmonic acid is activated by an enzyme that conjugates it to isoleucine in *Arabidopsis*. Plant Cell.

[32] Li Q., Zheng J., Li S., Huang G., Skilling S.J., Wang L., Li L., Li M., Yuan L., Liu P. (2017). Transporter-mediated nuclear entry of jasmonoyl-isoleucine is essential for jasmonate signaling. Mol. Plant.

[33] Thines B., Katsir L., Melotto M., Niu Y., Mandaokar A., Liu G., Nomura K., He S.Y., Howe G.A., Browse J. (2007). JAZ repressor proteins are targets of the SCF^COI1^ complex during jasmonate signalling. Nature.

[34] Fernández-Calvo P., Chini A., Fernández-Barbero G., Chico J.-M., Bimenez-Ibanez S., Geerinck J., Eeckhout D., Schweizer F., Godoy M., Franco-Zorrilla J.M. (2011). The Arabidopsis bHLH transcription factors MYC3 and MYC4 are targets of JAZ repressors and act additively with MYC2 in the activation of jasmonate responses. Plant Cell.

[35] Zhang F., Yao J., Ke J., Zhang L., Lam V.Q., Xin X.-F., Zhou X.E., Chen J., Brunzelle J., Griffin P.R. (2015). Structural basis of JAZ repression of MYC transcription factors in jasmonate signalling. Nature.

[36] Liu B., Seong K., Pang S., Song J., Gao H., Wang C., Zhai J., Zhang Y., Gao S., Li X. (2021). Functional specificity, diversity, and redundancy of *Arabidopsis* JAZ family repressors in jasmonate and COI1-regulated growth, development, and defense. New Phytol..

[37] Zhang C., Lei Y., Lu C., Wang L., Wu J. (2020). MYC2, MYC3, and MYC4 function additively in wounding-induced jasmonic acid biosynthesis and catabolism. J. Integr. Plant Biol..

[38] Glauser G., Dubugnon L., Mousavi S.A.R., Rudaz S., Wolfender J.-L., Farmer E.E. (2009). Velocity estimates for signal propagation leading to systemic jasmonic acid accumulation in wounded *Arabidopsis*. J. Biol. Chem..

[39] Schaller A., Stintzi A., Schaller A. (2008). Jasmonate biosynthesis and signaling for induced plant defense against herbivory. Induced Plant Resistance to Herbivores.

[40] Scholz S.S., Reichelt M., Boland W., Mithöfer A. (2015). Additional evidence against jasmonate-induced jasmonate induction hypothesis. Plant Sci..

[41] Thivierge K., Prado A., Driscoll B.T., Bonneil É., Thibault P., Bede J.C. (2010). Caterpillar- and salivary-specific modification of plant proteins. J. Proteome Res..

[42] Kaur D., Dorion S., Jmii S., Cappadocia L., Bede J.C., Rivoal J. (2023). Pseudophosphorylation of *Arabidopsis* jasmonate biosynthesis enzyme lipoxygenase 2 via mutation of SER^600^ inhibits enzyme activity. J. Biol. Chem..

[43] Sarde S.J., Kumar A., Remme R.N., Dicke M. (2018). Genome-wide identification, classification and expression of lipoxygenase gene family in pepper. Plant Mol. Biol..

[44] Kufel J., Diachenko N., Golisz A. (2022). Alternative splicing as a key player in the fine-tuning of the immunity response in *Arabidopsis*. Mol. Plant Pathol..

[45] Zhu J., Wang X., Guo L., Xu Q., Zhao S., Li F., Yan X., Liu S., Wei C. (2018). Characterization and alternative splicing profiles of the lipoxygenase gene family in tea plant (*Camellia sinensis*). Plant Cell Physiol..

[46] Moreno J.E., Shyu C., Campos M.L., Patel L.C., Chung H.S., Yao J., He S.Y., Howe G.A. (2013). Negative feedback control of jasmonate signaling by an alternative splice variant of JAZ10. Plant Physiol..

[47] Zhang F., Ke J., Zhang L., Chen R., Sugimoto K., Howe G.A., Xu H.E., Zhou M., He S.Y., Melcher K. (2017). Structural insights into alternative splicing-mediated desensitization of jasmonate signaling. Proc. Natl. Acad. Sci. USA.

[48] Nemchenko A., Kunze S., Feussner I., Kolomiets M. (2006). Duplicate maize 13-lipoxygenase genes are differentially regulated by circadian rhythm, cold stress, wounding, pathogen infection, and hormonal treatments. J. Exp. Bot..

[49] Weng J.-K., Chapple C. (2010). The origin and evolution of lignin biosynthesis. New Phytol..

[50] Vanholme R., Cesarino I., Rataj K., Xiao Y., Sundin L., Goeminne G., Kim H., Cross J., Morreel K., Araujo P. (2013). Caffeoyl shikimate esterase (CSE) is an enzyme in the lignin biosynthetic pathway in *Arabidopsis*. Science.

[51] Hirschmann F., Krause F., Papenbrock J. (2014). The multi-protein family of sulfotransferases in plants: Composition, occurrence, substrate specificity, and functions. Front. Plant Sci..

[52] Petersen M. (2016). Hydroxycinnamoyltransferases in plant metabolism. Phytochem. Rev..

[53] Yao T., Feng K., Xie M., Barros J., Tschaplinski T.J., Tuskan G.A., Muchero W., Chen J.-G. (2021). Phylogenetic occurrence of the phenylpropanoid pathway and lignin biosynthesis in plants. Front. Plant Sci..

[54] Zhou Y., Wu W., Sun Y., Shen Y., Mao L., Dai Y., Yang B., Liu Z. (2024). Integrated transcriptome and metabolome analysis reveals anthocyanin biosynthesis mechanisms in pepper (*Capsicum annuum* L.) leaves under continuous blue light irradiation. BMC Plant Biol..

[55] Mao Y., Luo J., Cai Z. (2025). Biosynthesis and regulatory mechanisms of plant flavonoids: A review. Plants.

[56] Berthet S., Thevenin J., Baratiny D., Demont-Caulet N., Debeaujon I., Bidzinski P., Leple J.-C., Huis R., Hawkins S., Gomez L.D. (2012). Role of plant laccases in lignin polymerization. Adv. Bot. Res..

[57] Lei G., Zhou K.-H., Chen X.-J., Huang Y.-Q., Yuan Z.-J., Li G.-G., Xie Y.-Y., Fang R. (2023). Transcriptome and metabolome analyses revealed the response mechanisms of pepper roots to *Phytophthora capsica* infection. BMC Genom..

[58] Oliveira D.M., Cesarino I. (2023). Finding my way: The role of dirigent proteins in lignin assembly. Mol. Plant.

[59] Koeda S., Noda T., Hachisu S., Kubo A., Tanaka Y., Yamamoto H., Ozaki S., Kinoshita M., Ohno K., Tanaka Y. (2023). Expression of alcohol acyltransferase in a potential determinant of fruit volatile ester variations in *Capsicum*. Plant Cell Rep..

[60] D’Auria J.C., Chen F., Pichersky E. (2002). Characterization of an acyltransferase capable of synthesizing benzylbenzoate and other volatile esters in flowers and damaged leaves of *Clarkia breweri*. Plant Physiol..

[61] Moura D.S., Ryan C.A. (2001). Wound-inducible proteinase inhibitors in pepper. Differential regulation upon wounding, systemin, and methyl jasmonate. Plant Physiol..

[62] Thomma B.P.H.J., Camme B.P.A., Thevissen K. (2002). Plant defensins. Planta.

[63] Mishra M., Mahajan N., Tamhane V.A., Kulkarni M., Baldwin I.T., Gupta V.S., Giri A.P. (2012). Stress inducible proteinase inhibitor diversity in *Capsicum annuum*. BMC Plant Biol..

[64] Heitz T., Widemann E., Lugan R., Miesch L., Ullmann P., Désaubry L., Holder E., Grausem B., Kandel S., Miesch M. (2012). Cytochromes P450 CYP94C1 and CYP94B3 catalyze two successive oxidation steps of plant hormone jasmonoyl-isoleucine for catabolic turnover. J. Biol. Chem..

[65] Koo A.J., Thireault C., Zemelis S., Poudel A.N., Zhang T., Kitaoka N., Brandizzi F., Matsuura H., Howe G.A. (2014). Endoplasmic reticulum-associated inactivation of the hormone jasmonoyl-l-isoleucine by multiple members of the cytochrome P450 94 family in Arabidopsis. J. Biol. Chem..

[66] Poudel A.N., Zhang T., Kwasniewski M., Nakabayashi R., Saito K., Koo A.J. (2016). Mutations in jasmonoyl-L-isoleucine-12-hydroxylases suppress multiple JA-dependent wound responses in *Arabidopsis thaliana*. Biochim. Biophys. Acta.

[67] Caarls L., Elberse J., Awwanah M., Ludwig N.R., de Vries M., Zeilmaker T., Van Wees S.C.M., Schuurink R.C., Van den Ackerveken G. (2017). *Arabidopsis* JASMONATE-INDUCED OXYGENASES down-regulate plant immunity by hydroxylation and inactivation of the hormone jasmonic acid. Proc. Natl. Acad. Sci. USA.

[68] Koo Y., Kim J.-J., Seo J.S., Kim J.-K., Choi Y.D. (2013). Characterization of methyl jasmonate specific esterase in Arabidopsis. J. Korean Soc. Appl. Biol. Chem..

[69] Wang X., Li N., Zan T., Xu K., Gao S., Yin Y., Yao M., Wang F. (2023). Genome-wide analysis of the TIFY family and function of *CaTIFY7* and *CaTIFY10b* under cold stress in pepper (*Capsicum annuum* L). Front. Plant Sci..

[70] Sarde S.J., Bouwmeester K., Venegas-Molina J., David A., Boland W., Dicke M. (2018). Involvement of Sweet Pepper *Calox2* in jasmonate-dependent induced defense against Western Flower Thrips. J. Integr. Plant Biol..

[71] Kim N., Lee J., Yeom S.-I., Kang N.-J., Kang W.-H. (2024). The landscape of abiotic and biotic stress-responsive splice variants with deep RNA-seq datasets in hot pepper. Sci. Data.

[72] De Vos M., Van Oosten V.R., Van Poeke R.M.P., Van Pelt J.A., Pozo M.J., Mueller M.J., Buchala A.J., Métraux J.-P., Van Loon L.C., Dicke M. (2005). Signal signature and transcriptome changes of Arabidopsis during pathogen and insect attack. Mol. Plant-Microbe Interact..

[73] Journot-Catalino N., Somssich I.E., Roby D., Kroj T. (2006). The transcription factors WRKY11 and WRKY17 act as negative regulators of basal resistance in *Arabidopsis thaliana*. Plant Cell.

[74] Pandey S.P., Roccaro M., Schön M., Logemann E., Somssich I.E. (2010). Transcriptional reprogramming regulated by WRKY18 and WRKY40 faciliates powdery mildew infection of Arabidopsis. Plant J..

[75] Kloth K.J., Wiegers G.L., Busscher-Lange J., van Haarst J.C., Kruijer W., Bouwmeester H.J., Dicke M., Jongsma M.A. (2016). AtWRKY22 promotes susceptility to aphids and modulates salicylic acid and jasmonic acid signaling. J. Exp. Bot..

[76] Jiao C., Li K., Zuo Y., Gong J., Guo Z., Shen Y. (2022). CALMODULIN1 and WRKY53 function in plant defense by negatively regulating jasmonic acid biosynthesis pathway in Arabidopsis. Int. J. Mol. Sci..

[77] Kandel S., Sauveplane V., Compagnon V., Franke R., Millet Y., Schreiber L., Werck-Reichhart D., Pinot F. (2007). Characterization of a methyl jasmonate and wounding-responsive cytochrome P450 of *Arabidopsis thaliana* catalyzing dicarboxylic fatty acid formation *in vitro*. FEBS J..

[78] Marquis V., Smirnova E., Graindorge S., Delcros P., Villette C., Zumsteg J., Heintz D., Heitz T. (2022). Broad-spectrum stress tolerance conferred by suppressing jasmonate signaling attenuation in Arabidopsis JASMONIC ACID OXIDASE mutants. Plant J..

[79] Zhang Y., Bouwmeester H.J., Kappers I.F. (2020). Combined transcriptome and metabolome analysis identifies defence responses in spider mite-infected pepper (*Capsicum annuum*). J. Exp. Bot..

[80] Zhao C.L., Cui X.M., Chen Y.P., Liang Q. (2010). Key enzymes of triterpenoid saponin biosynthesis and the induction of their activities and gene expressions in plants. Nat. Prod. Commun..

[81] Timmappa R., Geisler K., Louveau T., O’Maille P., Osbourn A. (2014). Triterpene biosynthesis in plants. Annu. Rev. Plant Biol..

[82] Seki H., Ohyama K., Sawai S., Mizutani M., Ohnishi T., Sudo H., Akashi T., Aoki T., Saito K., Muranaka T. (2008). Licorice β-amyrin 11-oxidase, a cytochrome P450 with a key role in the biosynthesis of the triterpene sweetener glycyrrhizin. Proc. Natl. Acad. Sci. USA.

[83] Asada K., Salim V., Masada-Atsumi S., Edmunds E., Nagatoshi M., Terasaka K., Mizukami H., De Luca V. (2013). A 7-deoxyloganetic acid glucosyltransferase contributes a key step in secologanin biosynthesis in Madagascar periwinkle. Plant Cell.

[84] von Steimker J., Wendenburg R., Klemmer A., Rosaria M., Fernie A.R., Alseekh S., Tripodi P. (2025). Genome-wide association analysis and linkage mapping decipher the genetic control of primary metabolites and quality traits in *Capsicum*. Plant J..

[85] Caputi L., Franke J., Farrow S.C., Chung K., Payne R.M.E., Nguyen T.-D., Dang T.-T.T., Soares Teto Carqueijeiro I., Koudounas K., de Bernonville T.D. (2018). Missing enzymes in the biosynthesis of the anticancer drug vinblastine in the Madagascar periwinkle. Science.

[86] Chowański S., Adamski Z., Marciniak P., Rosiński G., Büyükgüzel E., Büyükgüzel K., Falabella P., Scrano L., Ventrella E., Lelario F. (2016). A review of bioinsecticidal activity of *Solanaceae* alkaloids. Toxins.

[87] de Nijs M., Crews C., Dorgelo F., MacDonald S., Mulder P.P.J. (2023). Emerging issues on tropane alkaloid contamination of food in Europe. Toxins.

[88] Lee M.H., Jeon H.S., Kim S.H., Chung J.H., Roppolo D., Lee H.J., Cho H.J., Tobimatsu Y., Ralph J., Park O.K. (2019). Lignin-based barrier restricts pathogens to the infection site and confers resistance in plants. EMBO J..

[89] Ramoroson M.-L., Koutouan C., Helesbeaux J.-J., Le Clerc V., Hamama L., Geoffriau E., Briard M. (2022). Role of phenylpropanoids and flavonoids in plant resistance to pests and diseases. Molecules.

[90] Chezem W.R., Memon A., Li F.-S., Weng J.-K., Clay N.K. (2017). SG2-type R2R3-MYB transcription factor MYB15 controls defense-induced lignification and basal immunity in Arabidopsis. Plant Cell.

[91] Kim S.H., Lam P.Y., Lee M.-H., Jeon H.S., Tobimatsu Y., Park O.K. (2020). The Arabidopsis R2F3 MYB transcription factor MYB15 is a key regulator of lignin biosynthesis in effector-triggered immunity. Front. Plant Sci..

[92] Piontek K., Smith A.T., Blodig W. (2001). Lignin peroxidase structure and function. Biochem. Soc. Trans..

[93] Ayabi S., Akashi T. (2006). Cytochrome P450s in flavonoid metabolism. Phytochem. Rev..

[94] Zhang S., Yang J., Li H., Chiang V.L., Fu Y. (2021). Cooperative regulation of flavonoid and lignin biosynthesis in plants. Crit. Rev. Plant Sci..

[95] Teles Y.C.F., Souza M.S.R., de Fatima M., de Souza V. (2018). Sulfated flavonoids: Biosynthesis, structures and biological activities. Molecules.

[96] Bonde M.R., Millar R.L., Ingham J.L. (1973). Induction and identification of satival and vestitol as two phytoalexins from *Lotus corniculatus*. Phytochemistry.

[97] Wang X. (2011). Structure, function, and engineering of enzymes in isoflavonoid biosynthesis. Funct. Integr. Genom..

[98] Garcia-Calderón M., Pérez-Delgado C.M., Palove-Balang P., Betti M., Márquez A.J. (2020). Flavonoids and isoflavonoids biosynthesis in the model legume *Lotus japonicus*: Connections to nitrogen metabolism and photorespiration. Plants.

[99] Gupta A., Awasthi P., Sharma N., Parveen S., Vats R.P., Singh N., Kumar Y., Goel A., Chandran D. (2021). Medicarpin confers powdery mildew resistance in *Medicago truncatula* and activates the salicylic acid signalling pathway. Mol. Plant Pathol..

[100] Muroi A., Ishihara A., Tanaka C., Ishizuka A., Takabayashi J., Miyoshi H., Nishioka T. (2009). Accumulation of hydroxycinnamic acid amides induced by pathogen infection and identification of agmatine coumaroyltransferase in *Arabidopsis thaliana*. Planta.

[101] Morimoto N., Ueno K., Teraishi M., Okumoto Y., Mori N., Ishihara A. (2018). Induced phenylamide accumulation in response to pathogen infection and hormone treatment in rice (*Oryza sativa*). Biosci. Biotechnol. Biochem..

[102] Wang J., Song L., Gong X., Xu J., Li M. (2020). Functions of jasmonic acid in plant regulation and response to abiotic stress. Int. J. Mol. Sci..

[103] Han Z., Gao H., Ye L., Adil M.F., Ahsan M., Zhang G. (2023). Identification of QTLs associated with *p*-coumaric acid and ferulic acid in barley. Euphytica.

[104] Liu S., Jiang J., Ma Z., Xiao M., Yang L., Tian B., Yu Y., Bi C., Fang A., Yang Y. (2022). The role of hydroxycinnamic acid amide pathway in plant immunity. Front. Plant Sci..

[105] Xue R., Gao N., Chen J., Wu Z., Sun N., Li Y., Gong M., Zeng R., Song Y., Chen D. (2025). Hydroxycinnamic acid amides in rice: Biosynthesis, distribution, function, and implication for crop development. Front. Plant Sci..

[106] Turlings T.C.J., Erb M. (2018). Tritrophic interactions mediated by herbivore-induced plant volatiles: Mechanisms, ecological relevance, and application potential. Annu. Rev. Entomol..

[107] Li C., Zha W., Li W., Wang J., You A. (2023). Advances in the biosynthesis of terpenoids and their ecological functions in plant resistance. Int. J. Mol. Sci..

[108] Cardoza Y.J., Tumlinson J.H. (2006). Compatible and incompatible *Xanthomonas* infections differentially affect herbivore-induced volatile emission by pepper plants. J. Chem. Ecol..

[109] Choi H.W., Lee B.G., Kim N.H., Park Y., Lim C.W., Song H.K., Hwang B.K. (2008). A role for a methone reductase in resistance against microbial pathogens in plants. Plant Physiol..

[110] Gullner G., Künstler A., Király L., Pogány M., Tóbiás I. (2010). Up-regulated expression of lipoxygenase and divinyl ether synthase genes in pepper leaves inoculated with *Tobaoviruses*. Physiol. Mol. Plant Pathol..

[111] Fammartino A., Cardinale F., Göbel C., Mène-Saffrané L., Fournier J., Feussner I., Esquerré-Tugayé M.-T. (2007). Characterization of divinyl ether biosynthetic pathway specifically associated with pathogens in tobacco. Plant Physiol..

[112] Glauser G., Vallat A., Baldmer D. (2012). Hormone profiling. Methods Mol. Biol..

[113] Aranda P.S., LaJoie D.M., Jorcyk C.L. (2012). Bleach gel: A simple agarose gel for analyzing RNA quality. Electrophoresis.

[114] Andrews S., Krueger F., Segonds-Pichon A., Biggins L., Krueger C., Wingett S. (2010). FastQC: A Quality Control Tool for High Throughput Sequence Data. http://www.bioinformatics.babraham.ac.uk/projects/fastqc.

[115] Dobin A., Davis C., Schlesinger F., Drenkow J., Zaleski C., Jha S., Batut P., Chaisson M., Gingeras T.R. (2016). STAR: Ultrafast universal RNA-seq aligner. Bioinformatics.

[116] Love M.I., Huber W., Anders S. (2014). Moderated estimation of fold change and dispersion for RNA-seq data with DeSEQ2. Genome Biol..

[117] Liu P., Ewald J., Pang Z., Legrand E., Jeon Y.S., Sangiovanni J., Hacariz O., Zhou G., Head J.A., Basu N. (2023). ExpressAnalyst: A unified platform for RNA-sequencing analysis in non-model species. Nat. Commun..

[118] Ma P., Castillo-Davis C.I., Zhong W., Liu J.S. (2006). A data-driven clustering method for time course gene expression data. Nucleic Acids Res..

[119] Déjean S., Martin P.G., Baccini A., Besse P. (2007). Clustering time-series gene expression data using smoothing spline derivatives. EURASIP J. Bioinform. Syst. Biol..

[120] Wang Y., Xie Z., Kutschera E., Adams J.I., Kadash-Edmondson K.E., Xing Y. (2024). rMATS-turbo: An efficient and flexible computational tool for alternative splicing analysis of large-scale RNA-seq data. Nat. Protoc..

[121] Meng E.C., Goddard T.D., Pettersen E.F., Couch G.S., Pearson Z.J., Morris J.H., Ferrin T.E. (2023). UCSF ChimeraX: Tools for structure building and analysis. Protein Sci..

[122] Abramson J., Adler J., Dunger J., Evans R., Green T., Pritzel A., Ronneberger O., Willmore L., Ballard A.J., Bambrick J. (2024). Accurate structure prediction of biomolecular interactions with AlphaFold3. Nature.

[123] Oliveira Camargo P., Calzado N.F., Budzinski I.G.F., Domingues D.S. (2023). Genome-wide analysis of lipoxygenase (LOX) genes in angiosperms. Plants.

[124] Juhász C., Tóbiás I., Ádám A., Kátay G., Gullner G. (2015). Pepper 9- and 13-lipoxygenase genes are differentially activated by two tobamoviruses and by hormone treatments. Physiol. Mol. Plant Pathol..

